# Association of finerenone with graded proteinuria improvement in type 2 diabetes with chronic kidney disease and proteinuria >1 g/d: a retrospective cohort study

**DOI:** 10.3389/fendo.2026.1908577

**Published:** 2026-09-11

**Authors:** Na Sun, Xinyuan Song, Nan Hu, Jinping Li, Fengping Zhang, Wenxiu Chang

**Affiliations:** 1 Department of Nephrology, Tianjin First Central Hospital, Tianjin, China; 2 Department of Cell Transplantation, Tianjin First Central Hospital, Tianjin, China

**Keywords:** chronic kidney disease, finerenone, hyperkalemia, proteinuria, type 2 diabetes mellitus

## Abstract

**Background:**

Current evidence for finerenone in T2DM with CKD is derived primarily from patients with microalbuminuria to macroalbuminuria, and data in those with moderate-to-heavy proteinuria remain limited. This study aimed to investigate the effect of finerenone on sustained graded improvement of 24h-UTP and its safety profile in T2DM patients with CKD and proteinuria >1 g/d.

**Methods:**

This retrospective cohort study included patients with T2DM and CKD with proteinuria >1 g/d treated at hospital. Patients were divided into the finerenone group and the control group based on whether they received finerenone during the follow-up period. Proteinuria was graded as Stage 2 (1-3.5 g/d) and Stage 3 (>3.5 g/d). The primary endpoint was sustained graded improvement in 24h-UTP. The secondary endpoint was the percentage reduction in proteinuria from baseline at different timepoints. Propensity score weighting was applied to address baseline imbalances. Logistic regression and Kaplan-Meier analysis were used to assess the association between finerenone use and graded proteinuria improvement. A mixed model for repeated measures (MMRM) was employed to compare changes in 24h-UTP, eGFR, and serum potassium levels between the two groups. Safety outcomes included hyperkalemia incidence.

**Results:**

A total of 134 patients were included in this study, comprising 53 in the finerenone group and 81 in the control group. Over 12 months, 28 patients (52.8%) in the finerenone group and 27 patients (33.3%) in the control group achieved sustained graded improvement in 24h-UTP. After propensity score weighting, finerenone use was independently associated with sustained graded improvement (OR 1.26, 95% CI 1.07–1.47, P = 0.004). Kaplan-Meier analysis showed a significant difference in time to sustained improvement between groups (Log-rank P = 0.026-0.033). MMRM analysis revealed a greater reduction in 24h-UTP in the finerenone group, while eGFR and serum potassium changes did not differ significantly between groups.

**Conclusions:**

In patients with T2DM and proteinuria >1 g/d, finerenone increased the probability of sustained graded proteinuria improvement, shortened the time to improvement, and was not associated with a significantly increased risk of hyperkalemia over 12 months, although the interpretation of safety findings is limited by baseline differences in kidney function and concomitant medication use between groups.

## Introduction

Type 2 diabetes mellitus (T2DM) is a major public health concern worldwide, and its renal complications represent a significant source of morbidity and mortality (1). Proteinuria is a critical hallmark of renal disease progression in patients with T2DM, reflecting severe impairment of the glomerular filtration barrier. Persistent heavy proteinuria not only indicates glomerular damage but also functions as an independent risk factor that accelerates renal function deterioration and increases cardiovascular morbidity and mortality (2, 3). Reduction of proteinuria has been consistently associated with slowed chronic kidney disease (CKD) progression and improved renal outcomes, establishing it as a valid surrogate endpoint in clinical trials (4).

Although the combination of renin-angiotensin-aldosterone system inhibitors (RASis), including angiotensin-converting enzyme inhibitors (ACEIs) or angiotensin II receptor blockers (ARBs), with sodium-glucose cotransporter 2 inhibitors (SGLT2is) has become the standard of care for patients with T2DM and proteinuria, a substantial proportion of patients continue to experience persistent proteinuria and remain at high risk for renal and cardiovascular events (5, 6).

Mineralocorticoid receptor (MR) overactivation represents a key pathological mechanism driving kidney injury in T2DM with proteinuria. MR is widely distributed in renal podocytes, mesangial cells, fibroblasts, and tubular epithelial cells. Sustained MR activation, driven not only by aldosterone but also by cortisol-particularly under conditions of inflammation and oxidative stress prevalent in diabetic kidneys-promotes the release of proinflammatory cytokines, recruitment of inflammatory infiltrates, fibroblast proliferation, and podocyte apoptosis (7–9). In the context of significant proteinuria, MR expression is upregulated, creating a vicious cycle that exacerbates glomerulosclerosis and tubulointerstitial fibrosis, ultimately leading to irreversible renal function loss (10, 11). Notably, patients with more advanced CKD (stage 4) and heavy proteinuria appear to derive substantial benefit from finerenone, as demonstrated in a prespecified FIDELITY subgroup analysis showing consistent cardiorenal risk reduction across eGFR strata (10, 12). Finerenone, a novel nonsteroidal mineralocorticoid receptor antagonist (nsMRA), offers a targeted therapeutic approach by selectively blocking MR with high affinity, thereby inhibiting downstream inflammatory and fibrotic signaling pathways without the hormonal side effects associated with steroidal MRAs (13–15). The FIDELIO-DKD and FIGARO-DKD trials have demonstrated that finerenone significantly reduces proteinuria and improves cardiorenal outcomes in patients with T2DM and CKD (16–18). However, these landmark trials enrolled substantial proportions of patients with severe albuminuria, with 87% of participants in FIDELIO-DKD and 50% in FIGARO-DKD having UACR ≥300 mg/g at baseline (16, 17). Nevertheless, the inclusion criteria for both trials were based on UACR thresholds (≥30 to ≤300 mg/g for normoalbuminuria with diabetic retinopathy, or ≥300 mg/g for macroalbuminuria), and patients with more substantial proteinuria quantified by 24h-UTP (>1 g/d) were not specifically targeted. Consequently, evidence regarding the efficacy and safety of finerenone specifically in patients with T2DM and heavy proteinuria (24h-UTP >1 g/d) remains limited.

While UACR is the preferred measure for early diabetic kidney disease screening and cardiovascular risk stratification according to KDIGO guidelines, several considerations support the use of 24h-UTP in this study of patients with T2DM and moderate-to-heavy proteinuria. First, in patients with significant proteinuria, albumin becomes the dominant urinary protein and total proteinuria parallels albuminuria, rendering the advantage of albuminuria measurement over total proteinuria less pronounced (19). Second, the 1 g/day and 3.5 g/day cutoffs for 24h-UTP are well-established clinical thresholds with distinct prognostic significance in diabetic kidney disease. Zhou et al. demonstrated that nephrotic-range 24-hour proteinuria (≥3.5 g/day) was an independent predictor of poor renal survival in biopsy-proven diabetic nephropathy (HR 1.114, 95% CI 1.051–1.180, P<0.001), and was incorporated into a validated prognostic nomogram (20). Third, Minutolo et al. conducted a pooled analysis of four cohort studies in diabetic CKD, establishing that residual proteinuria levels stratify cardiorenal prognosis and that this prognostic value is independent of albuminuria-based classifications (21). Fourth, the 1 g/day threshold represents the transition to clinically significant proteinuria requiring further evaluation and often renal biopsy (22), and has been used as a treatment target in non-diabetic CKD, with meta-analyses showing that each 1 g/day reduction in proteinuria is associated with an 80% reduction in the risk of CKD progression or ESRD (23). The 3.5 g/day threshold defines nephrotic-range proteinuria, which is associated with distinct pathophysiological features including hypoalbuminemia, edema, and hyperlipidemia, and carries a significantly higher risk of rapid renal function decline and ESRD (2, 24). Fifth, in clinical practice, 24h-UTP remains the standard method for quantifying proteinuria in patients with substantial proteinuria, as validated by landmark trials including RENAAL and IDNT (25, 26). Finally, previous studies have demonstrated that in diabetic kidney disease with heavy proteinuria, total urine protein of 3.5 g/day is equivalent to urine albumin of 2.2 g/day, further validating the clinical relevance of total protein measurement in this population (27). These established prognostic thresholds, supported by recent evidence from biopsy-proven diabetic nephropathy cohorts and large pooled analyses, provide a clinically meaningful framework for assessing graded proteinuria improvement in patients with T2DM and baseline proteinuria >1 g/day.

This study aims to evaluate the efficacy and safety of finerenone in achieving sustained graded improvement of 24h-UTP in patients with T2DM and proteinuria >1 g/d, a population with more severe glomerular damage and higher cardiorenal risk but with limited evidence from prior randomized controlled trials. By focusing on this high-risk subgroup, we seek to address an important evidence gap and provide clinically relevant data to guide therapeutic decision-making in patients with significant persistent proteinuria.

## Materials and methods

### Study design and participants

This retrospective cohort study included patients with type 2 diabetes mellitus (T2DM) and proteinuria >1 g/d treated at Tianjin First Center Hospital between December 1, 2023, and November 30, 2024. Patients were divided into the finerenone group and the control group based on whether they received finerenone during the follow-up period. Treatment allocation was based on clinical decisions by the treating physicians rather than randomization, resulting in unequal group sizes (finerenone group, n=53; control group, n=81). This distribution reflects real-world clinical practice during the study period, when finerenone was recently introduced and prescribed selectively based on physician assessment of patient suitability.

The inclusion criteria were as follows: (1) diagnosis of T2DM according to the World Health Organization (WHO) or American Diabetes Association (ADA) criteria (28, 29); (2) baseline 24h-UTP >1 g/d; and (3) completion of 12-month follow-up with available clinical data. Non-diabetic kidney diseases were excluded using a combined diagnostic approach. Among the 134 patients included, 23 patients (17.2%) underwent renal biopsy, which confirmed diabetic nephropathy and ruled out other glomerular or tubulointerstitial diseases. For the remaining patients without biopsy, the diagnosis of diabetic kidney disease was established based on the following criteria: (1) a confirmed diagnosis of T2DM with a disease duration of ≥5 years; (2) the presence of diabetic retinopathy; and (3) the absence of clinical or laboratory features suggestive of non-diabetic kidney disease, including negative serological testing for autoimmune diseases (antinuclear antibody, anti-dsDNA antibody, antineutrophil cytoplasmic antibody) and normal serum complement levels (C3, C4), as well as normal findings on renal ultrasonography to exclude structural or obstructive renal diseases. Patients were further excluded if they had active urinary tract infection, nephrolithiasis, malignancy, or receipt of chemotherapy. Additionally, individuals with incomplete clinical records were excluded.

**Figure 1:**
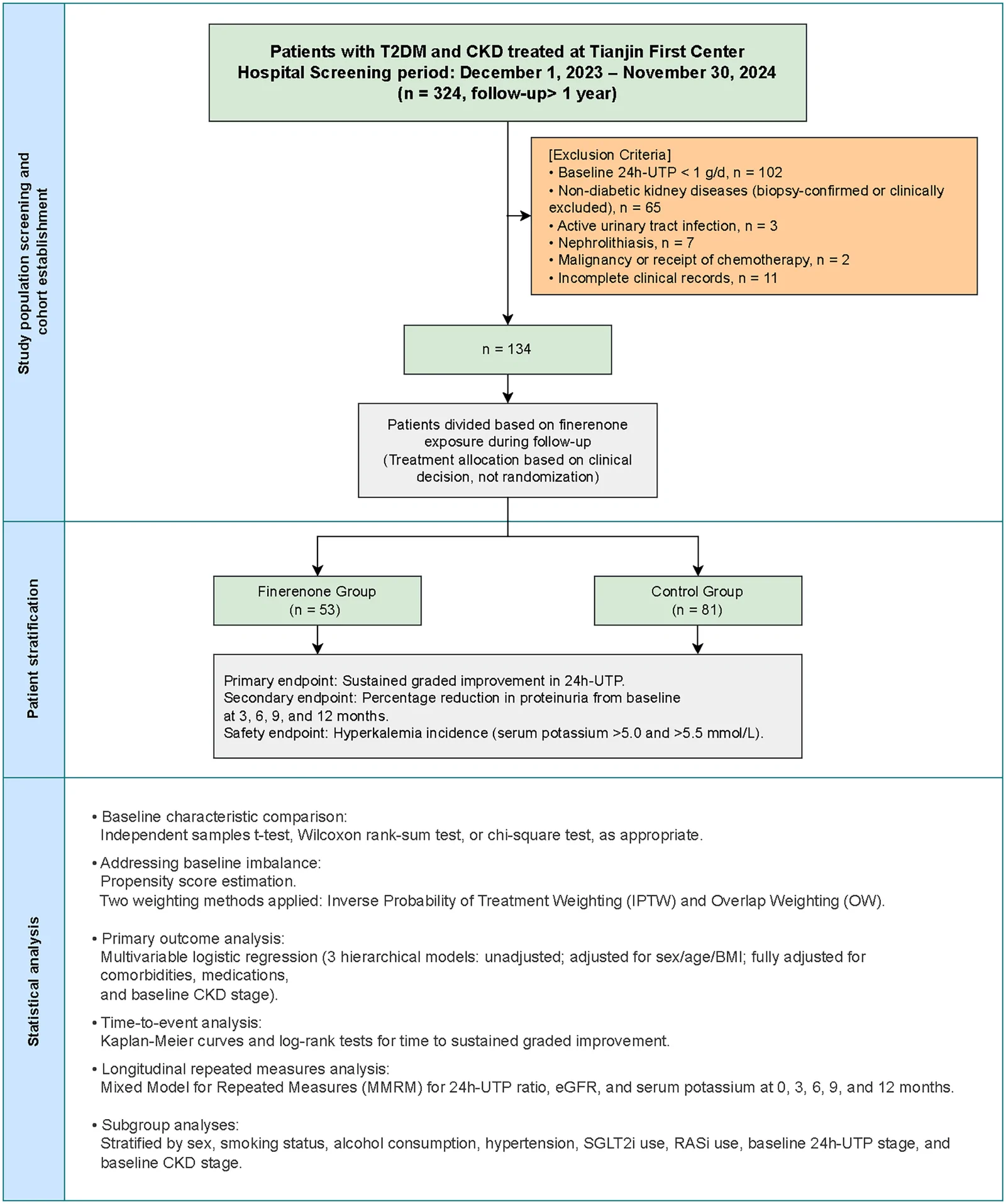
Study flow chart. T2DM, type 2 diabetes mellitus; CKD, chronic kidney disease; 24h-UTP, 24-hour urinary total protein. A total of 324 patients with T2DM and CKD were initially screened at Tianjin First Center Hospital between December 1, 2023, and November 30, 2024. After applying the exclusion criteria, 134 patients were included in the final analysis and divided into the finerenone group (n=53) and the control group (n=81).

A total of 324 patients with T2DM and CKD who were treated at Tianjin First Center Hospital during the study period (December 1, 2023, to November 30, 2024) were initially screened. Of these, 190 patients were excluded based on the following criteria: baseline 24h-UTP <1 g/d (n=102), non-diabetic kidney diseases confirmed by biopsy or clinically excluded (n=65), active urinary tract infection (n=3), nephrolithiasis (n=7), malignancy or receipt of chemotherapy (n=2), and incomplete clinical records (n=11). Ultimately, 134 patients met the eligibility criteria and were included in the final analysis (**Figure 1**).

The primary endpoint was sustained graded improvement in 24h-UTP, defined as a downgrade in proteinuria grade at any timepoint during follow-up that persisted until the end of the 12-month observation period. The secondary endpoint was the percentage reduction in proteinuria from baseline at different timepoints (3, 6, 9, and 12 months). Safety outcomes included the incidence of hyperkalemia during follow-up at 3, 6, 9, and 12 months, defined as serum potassium >5.0 mmol/L and >5.5 mmol/L, reported as both the number of patients and the number of episodes. This study was approved by the Ethics Committee of Tianjin First Center Hospital (approval number: 2015008S) and conducted in accordance with the principles of the Declaration of Helsinki. Written informed consent was waived by the Ethics Committee due to the retrospective nature of the study. All patient records and personal information were anonymized and de-identified to ensure data security and patient confidentiality.

### Sample size considerations

This was a retrospective observational study employing a consecutive enrollment principle, in which all eligible patients treated during the study period were included. Given that the sample was derived from an existing clinical database rather than prospectively recruited based on a pre-specified effect size, a traditional *a priori* sample size calculation was not performed. *Post-hoc* power analysis was conducted to characterize the statistical information of this study. Based on the observed event rates (finerenone group: 52.8%; control group: 33.3%), sample sizes (n=53 and n=81), and a two-sided α of 0.05, the *post-hoc* power for detecting the observed treatment effect (OR = 2.24) was approximately 60% by the two-proportion Z-test. The minimum detectable OR with 80% power under the current sample size was 2.73, indicating that the study may be underpowered for smaller effect sizes.

### Data collection

Data were extracted from the hospital electronic medical record system, encompassing general characteristics including sex, age, and body mass index (BMI); lifestyle factors such as smoking status (Yes=current or former smoker, No=never smoker) and alcohol consumption (Yes=current drinker, No=non-drinker); comorbidities including hypertension (defined as self-reported history, use of antihypertensive medication, or blood pressure ≥140/90 mmHg) and cardiovascular disease (including acute myocardial infarction, heart failure, coronary artery disease, and cerebral infarction); medication use including RASis, SGLT2is, and finerenone; and laboratory parameters including baseline and follow-up 24h-UTP, eGFR (calculated using the CKD-EPI equation) (30), serum potassium (measured by ion-selective electrode method), and fasting plasma glucose (measured by hexokinase method). All biochemical parameters were measured using an automated biochemical analyzer (cobas 701; Roche Diagnostics, Basel, Switzerland) in the central laboratory of Tianjin First Center Hospital. It is noted that not all patients in this cohort received guideline-recommended RASi or SGLT2i therapy (RASi use: 53.0%; SGLT2i use: 58.2%). The reasons for suboptimal medication use were multifactorial and included contraindications (e.g., eGFR below the threshold for SGLT2i initiation at the time of enrollment), drug intolerance (e.g., cough or angioedema with ACEI/ARB), economic constraints limiting drug accessibility, and individual physician clinical judgment regarding the risk-benefit balance in patients with advanced CKD or comorbidities.

### Definitions and grading criteria

For the grading criteria, 24h-UTP was classified into three stages using 1 g/d and 3.5 g/d as cutoffs: Stage 1 (<1 g/d, not included in this study), Stage 2 (1-3.5 g/d), and Stage 3 (>3.5 g/d). CKD stage was defined according to eGFR levels, categorized into five stages: Stage 1 (≥90 mL/min/1.73 m²), Stage 2 (60–89 mL/min/1.73 m²), Stage 3 (30–59 mL/min/1.73 m²), Stage 4 (15–29 mL/min/1.73 m²), and Stage 5 (<15 mL/min/1.73 m²). Sustained graded improvement was defined as a transition from a higher to a lower proteinuria stage that persisted without reversion to the original or a higher stage until the end of the 12-month follow-up.

### Statistical analysis

#### Data description and baseline comparisons

Continuous variables with normal distribution were expressed as mean ± standard deviation (SD), non-normally distributed continuous variables were presented as median (interquartile range) [M (Q1; Q3)], and categorical variables were described as frequency (percentage) [n (%)]. Group comparisons were performed using independent samples t-test, Wilcoxon rank-sum test, or chi-square test, as appropriate.

#### Study design and follow-up period

For the finerenone group, the observation baseline (t0) was defined as the date of finerenone initiation, with 12 months of subsequent follow-up. For the control group, the observation baseline was defined as the date of the first eligible visit, with 12 months of follow-up thereafter.

#### Finerenone treatment protocol

Finerenone was initiated at a dose of 5 mg or 10 mg once daily, with the starting dose selected based on baseline eGFR and serum potassium levels. Patients with lower eGFR or borderline serum potassium levels received the 5 mg starting dose, while those with relatively preserved kidney function and normal potassium levels initiated 10 mg once daily. Subsequent dose titration followed a stepwise protocol (5 mg to 10 mg to 20 mg once daily), with dose adjustments guided by periodic monitoring of eGFR and serum potassium.

Finerenone was discontinued or dose-reduced according to the following criteria: (1) serum potassium >5.0 mmol/L (dose reduction) or >5.5 mmol/L (discontinuation); (2) a decline in eGFR exceeding 30% from baseline (dose reduction or discontinuation at clinical discretion); and (3) patient intolerance or adverse events not manageable by dose adjustment. During dose reduction or temporary discontinuation, the decision to re-escalate or permanently discontinue finerenone was determined by the treating physician based on clinical reassessment.

### Addressing baseline imbalance: propensity score weighting

To address baseline imbalance inherent in this retrospective study design, propensity scores were estimated using multivariable logistic regression models incorporating the following measured covariates: age, sex, smoking history, alcohol consumption, BMI, history of hypertension, baseline fasting blood glucose, LDL-C, HDL-C, triglycerides, total cholesterol, HbA1c, baseline 24h-UTP, baseline eGFR, baseline SGLT2i use, and baseline RASi use. Both inverse probability of treatment weighting (IPTW) and overlap weighting (OW) were applied (31), and the calculated weights were assigned to each subject, thereby creating a pseudo-population in which baseline characteristics were independent of exposure status. Standardized mean differences (SMDs) were used to assess between-group balance before and after weighting, and all subsequent statistical analyses were performed using the newly formed weighted cohorts.

The selection of these covariates was based on the following considerations. CKD stage was chosen over continuous eGFR values based on the recommendations of the Kidney Disease: Improving Global Outcomes (KDIGO) 2012 CKD Guideline, which defines GFR categories (G1–G5) for CKD prognostic assessment (4). CKD stage integrates both eGFR levels and the clinical implications of renal function deterioration, providing a more clinically meaningful representation of baseline kidney disease severity than continuous eGFR values alone, and facilitating clearer clinical interpretation of baseline renal function distribution between groups. It is important to note that the KDIGO 2012 guideline employs albuminuria categories (A1-A3) based on UACR for proteinuria classification; however, the present study utilizes an independent 24h-UTP staging system (Stage 2: 1.0-3.5 g/24h; Stage 3: >3.5 g/24h) based on validated clinical prognostic thresholds, as detailed in the Introduction section. The 24h-UTP stage was selected over continuous eGFR values because the primary outcome of this study was defined as sustained graded improvement in 24h-UTP, which is categorized by proteinuria stages rather than absolute quantitative changes. The use of staged variables ensures consistency between baseline characteristic adjustment and outcome definition, thereby enhancing the clinical relevance and interpretability of the propensity score model. The remaining covariates-LDL-C, HDL-C, triglycerides, total cholesterol, and HbA1c-were selected based on their established associations with CKD progression and cardiorenal outcomes in patients with T2DM. Glycemic control (HbA1c) and lipid metabolism parameters are recognized modifiable risk factors for CKD progression and cardiovascular events, and their inclusion strengthens the adjustment for metabolic confounding. Blood pressure values (SBP and DBP) were added to **Tables 1** and **2** to provide a more comprehensive description of baseline cardiovascular risk profile, although SBP and DBP were not included as separate covariates in the propensity score model given that hypertension history (a composite indicator capturing both blood pressure levels and antihypertensive treatment) was already included. Creatinine was not included as a separate covariate because it is inherently captured through eGFR, which was derived from serum creatinine using the CKD-EPI equation; including both would introduce multicollinearity. Serum potassium was already included as a baseline covariate. Other potential confounders mentioned by the reviewer-including diabetes duration, waist-hip circumference, liver function enzymes, CRP, fasting insulin, UACR, serum sodium, and concomitant use of statins, diuretics, GLP-1RAs, or insulin-were not incorporated because these variables were either not routinely measured in this retrospective cohort, not reliably documented in the electronic medical records, or not applicable to this study population (e.g., UACR, given that 24h-UTP was the primary proteinuria measure). We have acknowledged these unmeasured confounders as a limitation in the Discussion section.

**Table 1:** Baseline characteristics of the study participants before propensity score weighting (N = 134).

Parameter	Total (N = 134)	Control group (N = 81)	Finerenone group (N = 53)	p.overall	SMD
Gender (Male, %)	102(76.1%)	63 (77.8%)	39 (73.6%)	0.727	0.098
Age (year)	54 [47;66]	54 [48;66]	53 [45;65]	0.512	0.108
BMI	26.01 [24.03;28.50]	26.12 [24.03;27.97]	25.95 [24.04;29.41]	0.831	0.114
SBP (mmHg)	135.50 [125.00, 144.75]	137.25 [126.41, 144.96]	129.88 [124.28, 144.67]	0.140	0.221
DBP (mmHg)	81.67 [79.00, 85.00]	81.90 [79.60, 85.00]	81.56 [78.40, 85.00]	0.525	0.053
Smoking history (Yes, %)	68 (50.7%)	41 (50.6%)	27 (50.9%)	1.000	0.007
Drinking history (Yes, %)	54 (40.3%)	36 (44.4%)	18 (34.0%)	0.303	0.216
Hypertension (Yes, %)	106 (79.1%)	67 (82.7%)	39 (73.6%)	0.292	0.222
Cardiovascular disease (Yes, %)	20 (14.9%)	12 (14.8%)	8 (15.1%)	1.000	0.00
Used SGLT2i (Yes, %)	78 (58.2%)	36 (44.4%)	42 (79.2%)	<0.001	0.767
Used RASi (Yes, %)	71 (53.0%)	36 (44.4%)	35 (66.0%)	0.023	0.445
HbA1c (%)	6.90 [6.20, 7.81]	6.86 [6.20, 8.29]	7.10 [6.00, 7.70]	0.718	0.150
LDL-C (mmol/L)	3.45 [2.56, 4.17]	3.44 [2.57, 4.08]	3.69 [2.55, 4.50]	0.255	0.167
HDL-C (mmol/L)	1.10 [0.91, 1.34]	1.08 [0.85, 1.30]	1.12 [0.99, 1.36]	0.129	0.150
Triglycerides (mmol/L)	1.75 [1.27, 2.54]	1.81 [1.17, 2.48]	1.68 [1.33, 2.58]	0.790	0.166
Total Cholesterol (mmol/L)	5.24 [4.23, 6.19]	5.13 [4.26, 5.97]	5.58 [4.21, 6.58]	0.149	0.144
Fasting Blood Glucose (mmol/L)	6.81 [5.76;8.43]	6.81 [5.81;8.61]	6.98 [5.75;8.41]	0.996	0.038
Serum Potassium (mmol/L)	4.37 [3.97;4.75]	4.50 [4.05;4.89]	4.30 [3.92;4.57]	0.137	0.259
eGFR (mL/min/1.73m²)	49.71 [32.21;79.18]	37.31 [28.64;62.60]	72.46 [44.21;92.30]	<0.001	0.802
24h-UTP (g/24h)	3.93 [2.01;6.47]	4.37 [2.84;7.06]	2.47 [1.66;5.25]	0.008	0.428
CKD stage				0.001	0.790
G1	22 (16.4%)	7 (8.6%)	15 (28.3%)		
G2	33 (24.6%)	16 (39.8%)	17 (32.1%)		
G3	48 (35.8%)	32 (39.5%)	16 (30.2%)		
G4	31 (23.1%)	26 (32.1%)	5 (9.4%)		
24h-UTP stages				0.024	0.440
1.0 ~ 3.5 g/24h	61 (45.5%)	30 (37.0%)	31 (58.5%)		
> 3.5 g/24h	73 (54.5%)	51 (63.0%)	22 (41.5%)		

Data are presented as median [interquartile range] for continuous variables with non-normal distribution, mean ± SD for normally distributed continuous variables, or as n (%) for categorical variables. The p.overall values were derived from Wilcoxon rank-sum test for non-normally distributed continuous variables, independent samples t-test for normally distributed continuous variables, and chi-square test for categorical variables, as appropriate. SMDs were calculated to assess baseline balance between groups. CKD stages were defined according to eGFR levels: G1 (≥90 mL/min/1.73 m²), G2 (60–89 mL/min/1.73 m²), G3 (30–59 mL/min/1.73 m²), and G4 (15–29 mL/min/1.73 m²). 24h-UTP stages were classified as 1.0-3.5 g/24h and >3.5 g/24h.

BMI, body mass index; SBP, systolic blood pressure; DBP, diastolic blood pressure; HbA1c, glycated hemoglobin A1c; LDL-C, low-density lipoprotein cholesterol; HDL-C, high-density lipoprotein cholesterol; SGLT2i, sodium-glucose cotransporter 2 inhibitor; RASi, renin-angiotensin-aldosterone system inhibitor; eGFR, estimated glomerular filtration rate; CKD, chronic kidney disease; 24h-UTP, 24-hour urinary total protein; SMD, standardized mean difference.

**Table 2:** Baseline characteristics of the study participants after propensity score weighting.

Parameter	IPTW-weighted				OW-weighted			
	Control group N=81	Finerenone group N=53	SMD	p.overall	Control group N=81	Finerenone group N=53	SMD	p.overall
Gender (Male,%)	63.86 (78.84%)	42.76 (80.68%)	0.043	0.809	15.98 (19.72%)	10.45 (19.72%)	0	1
Age(year)	54.00 (48.00, 64.00)	55.00 (45.00, 67.00)	-0.05	0.728	54.00 (47.00, 63.00)	54.00 (44.00, 65.00)	0	0.902
BMI	26.44 (24.06, 29.11)	26.20 (23.43, 29.91)	0.079	0.817	26.12 (24.06, 29.07)	26.20 (23.43, 29.41)	0	0.995
SBP (mmHg)	135.50 (126.38, 144.20)	129.88 (125.00, 145.83)	0.006	0.716	135.00 (126.00, 144.20)	129.88 (125.00, 145.00)	0	0.775
Smoking history (Yes, %)	39.52 (48.79%)	26.99 (50.92%)	0.043	0.841	41.87 (51.69%)	27.39 (51.69%)	0	1
Drinking history (Yes, %)	32.55 (40.19%)	19.86 (37.47%)	0.056	0.793	30.64 (37.83%)	20.05 (37.83%)	0	1
Used SGLT2i (Yes, %)	47.25 (58.34%)	35.44 (66.86%)	0.188	0.427	55.83 (68.93%)	36.53 (68.93%)	0	1
Used RASi (Yes, %)	44.71 (55.20%)	31.20 (58.87%)	0.076	0.727	49.11 (60.62%)	32.13 (60.62%)	0	1
Fasting Blood Glucose (mmol/L)	7.12 (6.17, 8.79)	6.98 (6.18, 8.41)	0.07	0.737	7.12 (6.24, 7.96)	6.98 (6.18, 8.41)	0	0.803
Serum Potassium (mmol/L)	4.46 (3.90, 4.80)	4.34 (3.91, 4.57)	0.067	0.663	4.43 (3.90, 4.74)	4.34 (3.92, 4.57)	0	0.905
eGFR (mL/min/1.73m²)	54.82 (32.38, 67.10)	64.32 (39.92, 86.96)	-0.271	0.203	60.21 (37.42, 82.50)	64.32 (39.92, 85.41)	0	0.894
24h-UTP (g/24h)	3.64 (2.24, 5.90)	3.40 (1.73, 5.77)	0.052	0.742	3.41 (2.24, 5.58)	3.40 (1.78, 5.60)	0	0.874
LDL-C (mmol/L)	3.43 (2.57, 4.06)	3.42 (2.47, 4.44)	-0.061	0.539	3.43 (2.57, 4.12)	3.69 (2.61, 4.45)	0	0.547
HDL-C (mmol/L)	1.05 (0.83, 1.25)	1.08 (0.98, 1.35)	-0.088	0.303	1.13 (0.85, 1.34)	1.14 (0.98, 1.39)	0	0.526
Triglycerides (mmol/L)	1.70 (1.26, 2.68)	1.64 (1.31, 2.26)	-0.044	0.948	1.66 (1.11, 2.77)	1.64 (1.33, 2.29)	0	0.937
Total Cholesterol (mmol/L)	5.02 (4.31, 5.96)	5.07 (4.09, 6.58)	-0.039	0.618	5.01 (4.31, 5.98)	5.29 (4.09, 6.58)	0	0.622
HbA1c (%)	6.77 (6.30, 8.28)	7.10 (6.20, 7.70)	0.067	0.914	6.74 (6.30, 8.28)	6.90 (6.20, 7.70)	0	0.928

Data are presented as weighted median (interquartile range) for continuous variables, or as weighted n (%) for categorical variables. The p.overall values and SMDs were calculated after propensity score weighting. For IPTW weighting, most covariates achieved adequate balance (SMD <0.1), with the exception of SGLT2i use (SMD = 0.188) and eGFR (SMD=-0.271), which were further adjusted in the regression models. For OW weighting, all covariates achieved excellent balance (all SMDs <0.001). CKD stages were defined according to eGFR levels: G1 (≥90 mL/min/1.73 m²), G2 (60–89 mL/min/1.73 m²), G3 (30–59 mL/min/1.73 m²), and G4 (15–29 mL/min/1.73 m²). 24h-UTP stages were classified as 1.0–3.5 g/24h and >3.5 g/24h.

IPTW, inverse probability of treatment weighting; OW, overlap weighting; SBP, systolic blood pressure; HbA1c, glycated hemoglobin A1c; LDL-C, low-density lipoprotein cholesterol; HDL-C, high-density lipoprotein cholesterol; SGLT2i, sodium-glucose cotransporter 2 inhibitor; RASi, renin-angiotensin-aldosterone system inhibitor; eGFR, estimated glomerular filtration rate; 24h-UTP, 24-hour urinary total protein; SMD, standardized mean difference.

### Primary outcome: association with sustained graded improvement in 24h-UTP

Multivariable logistic regression was used to assess the association between finerenone use and sustained graded improvement in 24h-UTP. Three hierarchical models were constructed: Model 1 included only the group variable (finerenone use); Model 2 additionally adjusted for sex, age, and BMI; and Model 3 further adjusted for comorbidities (hypertension, cardiovascular disease), concomitant medications (RASis, SGLT2is), and baseline CKD stage. For the IPTW and overlap-weighted cohorts, models were built incorporating the group variable (finerenone use) and all covariates with SMD > 0.1.

### Time-to-event analysis

Kaplan-Meier curves and log-rank tests were employed to evaluate the association between finerenone use and time to sustained graded improvement in 24h-UTP. Kaplan-Meier analysis was used as the sole time-to-event method because all patients completed the 12-month follow-up period (minimizing censoring), and the limited sample size precluded multivariable Cox proportional hazards regression due to insufficient events per covariate. KM analysis, combined with logistic regression for the binary outcome and MMRM for longitudinal trajectories, provided a complementary analytical framework covering the probability, timing, and trajectory of treatment response.

### Longitudinal analysis: repeated measures over time

MMRM was used to analyze the temporal changes in 24h-UTP between the two groups. Specifically, changes in the ratio of 24h-UTP to baseline at 3, 6, 9, and 12 months were analyzed, with age, sex, RASi use, SGLT2i use, baseline CKD stage, and baseline 24h-UTP stage included as covariates. An interaction term between treatment group and timepoint was incorporated to assess how the between-group effect varied over time. Subjects were treated as random effects, and a first-order ante-dependence (AD) covariance structure was specified. Model parameters were estimated using maximum likelihood (ML). The same model structure was applied to further analyze the temporal trends of eGFR and serum potassium.

### Subgroup analyses

Subgroup analyses were conducted according to sex, smoking status, alcohol consumption, hypertension, concomitant medication use (SGLT2is, RASis), baseline 24h-UTP stage, and baseline CKD stage to explore the consistency of finerenone efficacy across different patient characteristics.

### Missing data and statistical software

Complete case analysis was employed, and multiple imputation was not performed. All statistical analyses were conducted using R software version 4.6.0 A two-sided P-value <0.05 was considered statistically significant.

## Results

### Baseline characteristics of study participants

A total of 324 patients were initially screened, of whom 134 met the inclusion criteria and were enrolled in this study (**Figure 1**), comprising 81 in the control group and 53 in the finerenone group. The overall median age was 54.0 [47, 66] years, with 102 (76.1%) males. The median baseline 24h-UTP was 3.93 [2.01; 6.47] g/24h, baseline eGFR was 49.71 [32.21; 79.18] mL/min/1.73m², baseline fasting plasma glucose was 6.81 [5.76; 8.43.] mmol/L, and baseline serum potassium was 4.37 [3.97; 4.75] mmol/L.

Before propensity score weighting, significant baseline imbalances were observed between the two groups (**Table 1**). The finerenone group had significantly higher baseline eGFR levels compared to the control group (72.46 [44.21; 92.30] vs. 37.31 [28.64; 62.60] mL/min/1.73m², P<0.001), with corresponding differences in CKD stage distribution (P = 0.001). The finerenone group also had lower baseline 24h-UTP levels (2.47 [1.66; 5.25] vs. 4.37 [2.84; 7.06] g/24h, P = 0.008) and a higher proportion of patients with 24h-UTP in the 1.0-3.5 g/24h range (58.5% vs. 37.0%, P = 0.024). Additionally, the use of SGLT2is (79.2% vs. 44.4%, P<0.001) and RASis (66.0% vs. 44.4%, P = 0.023) was significantly more frequent in the finerenone group. No significant differences were observed between groups in terms of sex, age, BMI, smoking history, drinking history, hypertension, cardiovascular disease, fasting plasma glucose, or serum potassium levels (all P>0.05).

After propensity score weighting, the balance of baseline characteristics improved substantially for most covariates (**Table 2**). However, certain variables, including baseline eGFR and SGLT2i use, retained SMD values exceeding 0.1 after IPTW weighting, indicating mild residual imbalance. The OW weighting method achieved better balance for most variables compared with IPTW. To address the residual confounding from persistently imbalanced variables, subsequent regression analyses incorporated additional adjustment for these covariates (see Statistical Analysis section).

### Logistic regression analysis of sustained graded improvement in 24h-UTP

During the 12-month follow-up period, a total of 55 patients (41.04%) achieved sustained graded improvement in 24h-UTP. In the finerenone group (n=53), 28 patients (52.8%) achieved sustained graded improvement, including 13 patients (24.5%) who improved from grade 2 to grade 1, 11 patients (20.8%) who improved from grade 3 to grade 2, and 4 patients (7.5%) who improved from grade 3 to grade 1. In the control group (n=81), 27 patients (33.3%) achieved sustained graded improvement, including 7 patients (8.6%) who improved from grade 2 to grade 1, 15 patients (18.5%) who improved from grade 3 to grade 2, and 5 patients (6.2%) who improved from grade 3 to grade 1. Overall, 26 patients improved from grade 3 to grade 2, 20 patients from grade 2 to grade 1, and 9 patients from grade 3 to grade 1.

In the unweighted cohort (**Table 3**), multivariable logistic regression analysis showed that finerenone use was significantly associated with sustained graded improvement in 24h-UTP in Model 1 (unadjusted: OR = 2.24, 95% CI = 1.11-4.60, P = 0.026) and Model 2 (adjusted for age, sex, and BMI: OR = 2.24, 95% CI = 1.10-4.64, P = 0.027). However, after full adjustment for comorbidities (hypertension, cardiovascular disease), concomitant medications (RASis, SGLT2is), baseline CKD stage, and baseline 24h-UTP stage in Model 3, the association was attenuated and no longer statistically significant (OR = 2.10, 95% CI = 0.90-5.05, P = 0.089).

**Table 3:** Logistic regression analysis of finerenone application and sustained improvement in 24h-UTP grading.

Unweighted	OR (95% CI)	P value
Model 1: Unadjusted	2.24 (1.11-4.60)	0.026
Model 2: Adjusted for age, sex, BMI	2.24 (1.10-4.64)	0.027
Model 3: Fully adjusted	2.10 (0.90-5.05)	0.089
IPTW-weighted		
Model 1: Unadjusted	1.29 (1.11-1.50)	0.000
Model 2: Adjusted for SGLT2i, baseline eGfr	1.26 (1.07 -1.47)	0.004
OW-weighted		
Model 1: Unadjusted	1.25 (1.09-1.43)	0.001

OR, odds ratio; CI, confidence interval; IPTW, inverse probability of treatment weighting; OW, overlap weighting; BMI, body mass index; RASi, renin-angiotensin-aldosterone system inhibitor; SGLT2i, sodium-glucose cotransporter 2 inhibitor; 24h-UTP, 24-hour urinary total protein.

Unweighted cohort: Model 1=unadjusted; Model 2=adjusted for age, sex, BMI; Model 3=fully adjusted (additionally for hypertension, cardiovascular disease, RASis, SGLT2is, baseline CKD stage, and 24h-UTP stage). IPTW-weighted cohort: Model 1=unadjusted; Model 2=adjusted for SGLT2i use and baseline eGFR (variables with residual SMD >0.1). OW-weighted cohort: Model 1 only (all SMDs <0.001, no additional adjustment needed). Primary outcome: sustained graded improvement in 24h-UTP persisted until 12-month follow-up.

After propensity score weighting (**Table 3**), the association between finerenone use and sustained graded improvement in 24h-UTP remained significant across all three models. In the IPTW-weighted cohort (**Table 3**), finerenone use was significantly associated with sustained graded improvement in 24h-UTP in Model 1 (unadjusted: OR = 1.29, 95% CI = 1.11-1.50, P<0.001) and Model 2 (additionally adjusted for SGLT2i use and baseline eGFR: OR = 1.26, 95% CI = 1.07-1.47, P = 0.004). In the OW-weighted cohort, finerenone use was also significantly associated with the outcome in Model 1 (OR = 1.25, 95% CI = 1.09-1.43, P = 0.001).

The consistency of significant findings across all three analytical approaches - unweighted, IPTW-weighted, and OW-weighted - supports the robustness of the association between finerenone use and sustained graded improvement in 24h-UTP. The additional adjustment for residual confounders (baseline eGFR and SGLT2i use) in the IPTW-weighted Model 2 further strengthened confidence in this finding by addressing the remaining baseline imbalance. This suggests that baseline confounding, particularly the imbalances in eGFR, CKD stage, 24h-UTP stage, and concomitant medication use, may have masked the true treatment effect in the unweighted cohort. The consistency of results across both IPTW and OW weighting methods further supports the robustness of this finding.

### Kaplan-Meier analysis and log-rank test for time to sustained graded improvement in 24h-UTP

Kaplan-Meier analysis was performed to evaluate the time to sustained graded improvement in 24h-UTP across three analytical cohorts: unweighted, IPTW-weighted, and OW-weighted.

**Figure 2:**
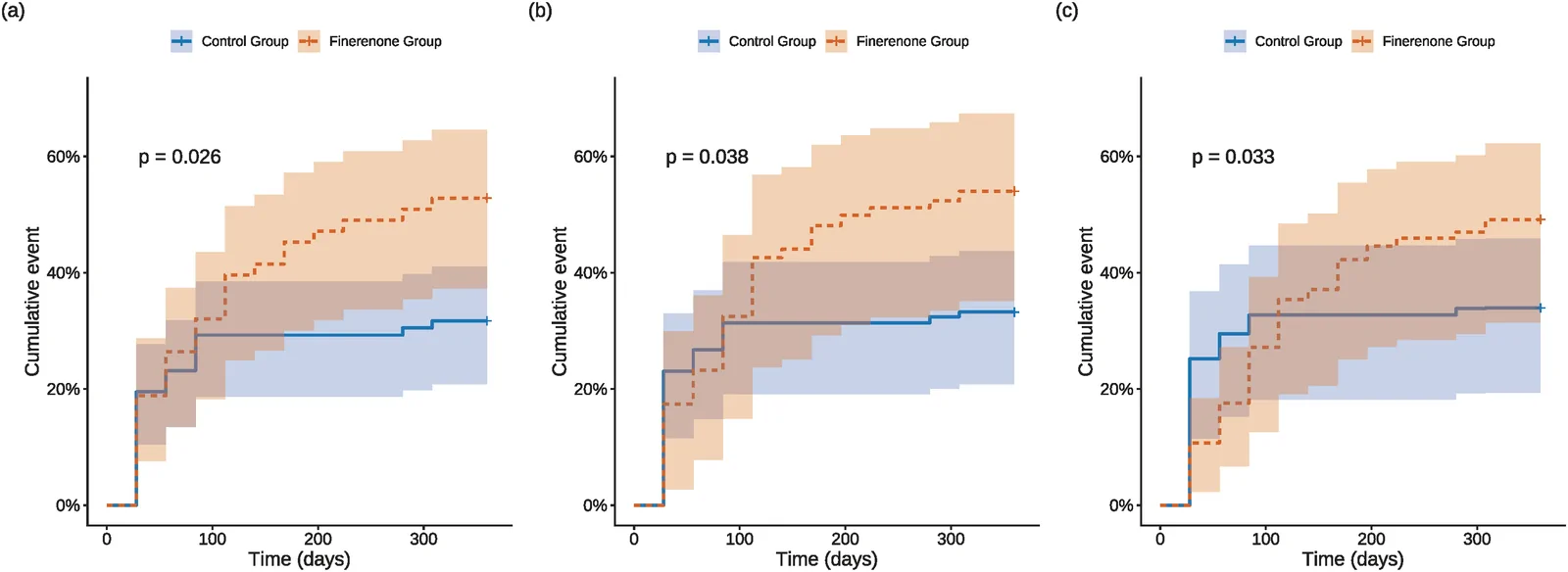
Kaplan-Meier curves for time to sustained graded improvement in 24h-UTP. **(a)** Unweighted cohort; **(b)** IPTW-weighted cohort; **(c)** OW-weighted cohort. Log-rank P = 0.026, 0.038, and 0.033, respectively.

In the unweighted cohort (**Figure 2A**), the cumulative incidence of sustained graded improvement in 24h-UTP was consistently higher in the finerenone group compared to the control group throughout the 12-month follow-up period. The finerenone group demonstrated a steeper early rise in cumulative event rate, reaching approximately 50% by 12 months, whereas the control group plateaued at approximately 33%. Log-rank test revealed a statistically significant difference between the two groups (P = 0.026).

In the IPTW-weighted cohort (**Figure 2B**), after adjusting for baseline imbalances through inverse probability of treatment weighting, the finerenone group maintained a higher cumulative incidence of sustained graded improvement in 24h-UTP compared to the control group. The separation between the two curves was evident from early follow-up and persisted throughout the observation period, with the finerenone group reaching approximately 50% cumulative event rate versus approximately 33% in the control group at 12 months. The log-rank test remained statistically significant (P = 0.038).

In the OW-weighted cohort (**Figure 2C**), similar findings were observed after overlap weighting. The finerenone group showed a higher cumulative incidence of sustained graded improvement in 24h-UTP compared to the control group, with the two curves diverging early and maintaining separation throughout follow-up. The 12-month cumulative event rates were approximately 50% in the finerenone group and 33% in the control group. The log-rank test again demonstrated a statistically significant difference between groups (P = 0.033).

The consistency of significant log-rank test results across all three analytical approaches-unweighted, IPTW-weighted, and OW-weighted-supports the robustness of the finding that finerenone use is associated with a significantly shorter time to sustained graded improvement in 24h-UTP compared to the control group. Notably, the P-values remained significant across all three weighting strategies (unweighted: P = 0.026; IPTW-weighted: P = 0.038; OW-weighted: P = 0.033), suggesting that the observed treatment effect on time-to-event outcome is not substantially confounded by the measured baseline imbalances.

### Subgroup analysis

**Figure 3:**
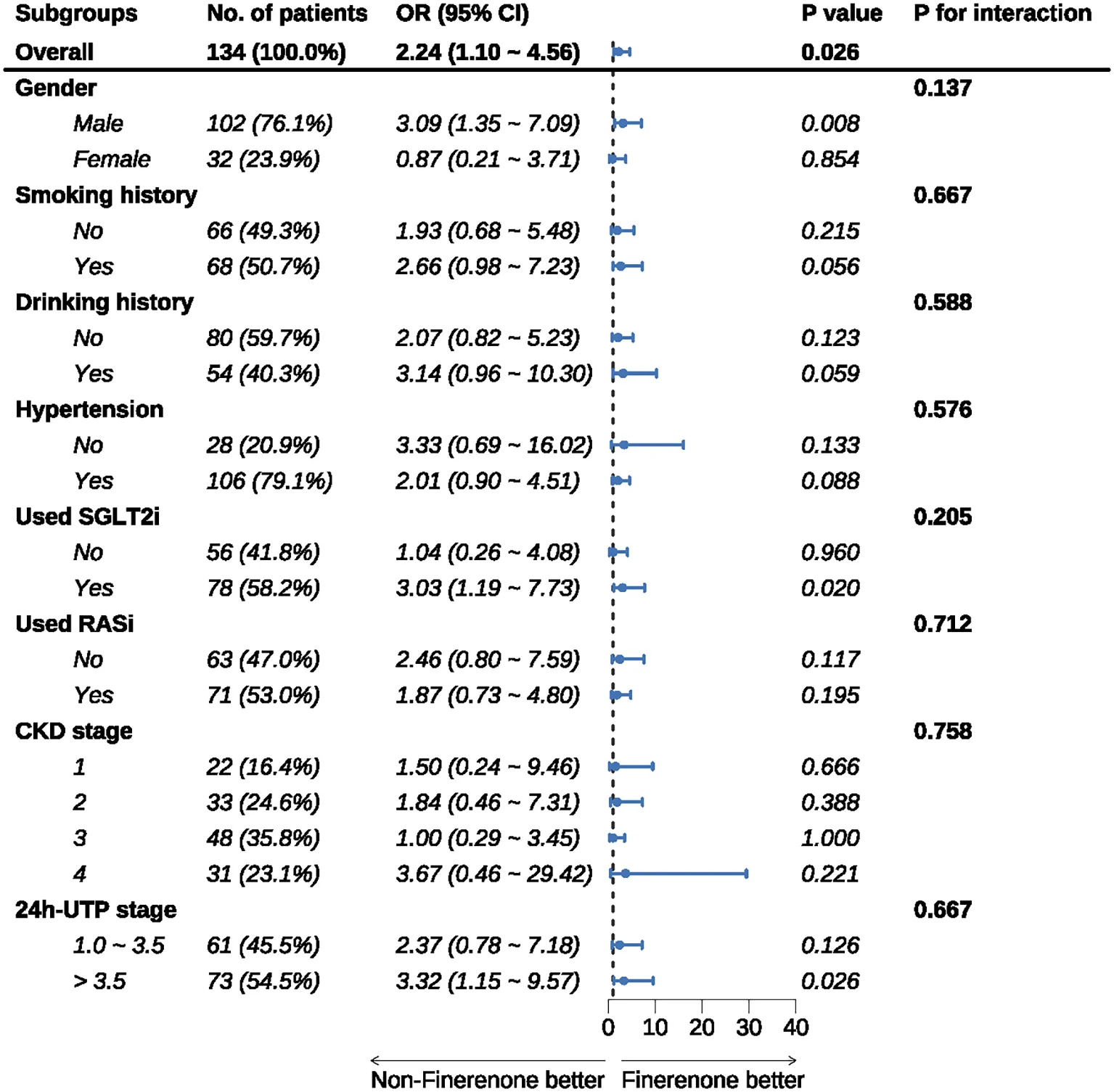
Subgroup analyses of the association between finerenone use and sustained graded improvement in 24h-UTP. ORs and 95% CIs are shown for each subgroup. P values for interaction are provided where applicable.

Subgroup analyses were conducted in the unweighted cohort to explore the consistency of finerenone efficacy across different patient characteristics (**Figure 3**). Overall, finerenone use was significantly associated with sustained graded improvement in 24h-UTP (OR = 2.24, 95% CI = 1.10-4.56, P = 0.026). Across sex subgroups, the point estimates of OR differed numerically (males: OR = 3.09, 95% CI = 1.35-7.09; females: OR = 0.87, 95% CI = 0.21-3.71); however, the interaction test was not statistically significant (P for interaction=0.137), indicating that this numerical difference does not reach statistical significance. Among patients receiving concomitant SGLT2i therapy, finerenone use was associated with sustained graded improvement (OR = 3.03, 95% CI = 1.19-7.73), while in those without SGLT2i use, the association was not statistically significant (OR = 1.04, 95% CI = 0.26-4.08); however, the interaction test was also not significant (P for interaction=0.205). Similarly, in patients with baseline 24h-UTP >3.5 g/24h, the OR was 3.32 (95% CI = 1.15-9.57), and in those with 24h-UTP 1.0-3.5 g/24h, the OR was 2.37 (95% CI = 0.78-7.18), with no significant interaction between subgroups (P for interaction=0.667). No significant treatment effects or interactions were observed across subgroups defined by smoking history, drinking history, hypertension, RASi use, or CKD stage (all P for interaction >0.05). These subgroup analyses are exploratory in nature. Given that none of the interaction tests reached statistical significance, the observed numerical differences in point estimates across subgroups should not be interpreted as evidence of differential treatment effects. The limited sample size within certain subgroups likely resulted in insufficient statistical power to detect true interaction effects.

### MMRM analysis of longitudinal changes

#### Temporal trends in 24h-UTP

**Figure 4:**
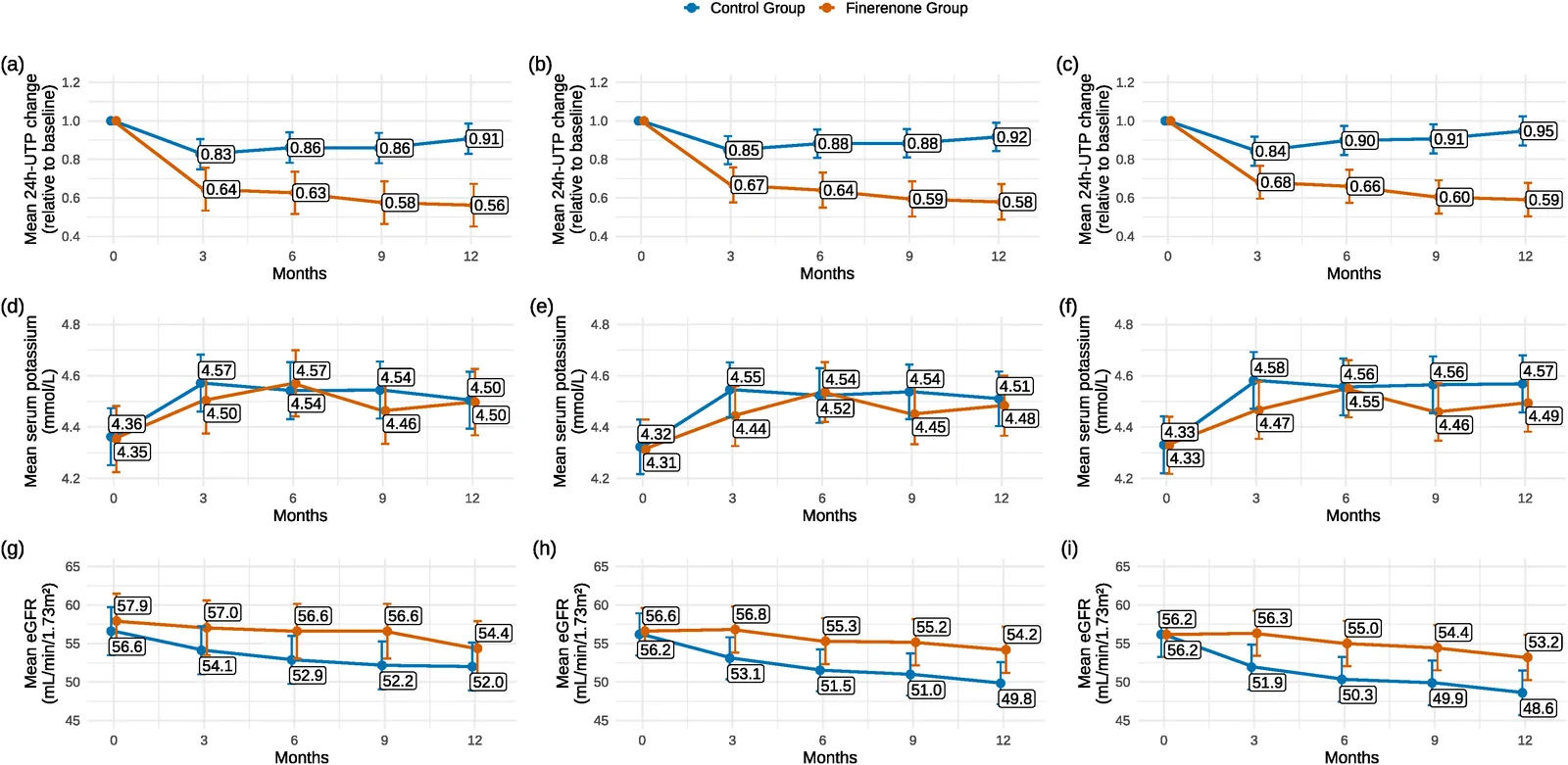
Temporal changes in clinical parameters by MMRM analysis across three analytical cohorts. **(a-c)** 24h-UTP ratio: **(a)** Unweighted; **(b)** IPTW-weighted; **(c)** OW-weighted. The finerenone group consistently showed lower 24h-UTP ratios at all post-baseline timepoints. **(d-f)** Serum potassium trajectories and **(g-i)** eGFR trajectories in the corresponding cohorts. Both eGFR and serum potassium remained stable in both groups, with no significant between-group differences in temporal trends (all P>0.05).

MMRM analysis was used to evaluate the longitudinal trajectory of 24h-UTP relative to baseline across three cohorts (**Figure 4**). At baseline (day 0), both groups had an adjusted 24h-UTP ratio of 1.0. In the unweighted cohort (**Figure 4A**), the finerenone group demonstrated a marked and sustained reduction in 24h-UTP ratio throughout follow-up, declining from 1.0 at baseline to 0.64 at 3 months, 0.63 at 6 months, 0.58 at 9 months, and 0.56 at 12 months. In contrast, the control group showed minimal reduction, with ratios of 0.83, 0.86, 0.86, and 0.91 at the corresponding timepoints, and even exhibited a slight increase from month 9 to month 12. The between-group divergence was evident from the first follow-up assessment and progressively widened over time.

Similar patterns were observed in the IPTW-weighted cohort (**Figure 4B**) and OW-weighted cohort (**Figure 4C**). In the IPTW-weighted analysis, the finerenone group achieved 24h-UTP ratios of 0.67, 0.64, 0.59, and 0.58 at 3, 6, 9, and 12 months, respectively, while the control group maintained higher ratios of 0.85, 0.88, 0.88, and 0.92. The OW-weighted analysis yielded comparable results, with finerenone group ratios of 0.68, 0.66, 0.60, and 0.59, versus control group ratios of 0.84, 0.90, 0.91, and 0.95.

Across all three analytical approaches, the finerenone group consistently showed lower 24h-UTP ratios than the control group at every post-baseline timepoint, with the magnitude of between-group difference increasing over time. This indicates that finerenone use is associated with a more pronounced and sustained reduction in proteinuria levels compared to standard care. The consistency of these findings across unweighted and weighted cohorts further supports the robustness of the treatment effect.

#### Temporal trends in serum potassium

MMRM analysis was further applied to assess the longitudinal trajectory of serum potassium levels across the three cohorts (**Figures 4D–F**). In the unweighted cohort (**Figure 4D**), serum potassium levels in both groups remained within the normal range throughout the follow-up period, with mean values fluctuating between 4.35 and 4.57 mmol/L in the finerenone group and between 4.35 and 4.57 mmol/L in the control group. Similar patterns were observed in the IPTW-weighted (**Figure 3E**) and OW-weighted cohorts (**Figure 4F**). Across all three analytical approaches, serum potassium levels remained stable in both groups, and no significant differences in temporal trends were observed between groups (all P>0.05), indicating that finerenone was not associated with an increased risk of hyperkalemia or significant electrolyte disturbances.

#### Temporal trends in eGFR

MMRM analysis was also used to evaluate the longitudinal trajectory of eGFR across the three cohorts (**Figures 4G–I**). In the unweighted cohort (**Figure 4G**), the finerenone group showed relatively stable eGFR levels throughout the follow-up period, with mean values ranging from 57.8 mL/min/1.73 m² at baseline to 54.4 mL/min/1.73 m² at 12 months, while the control group exhibited a gradual decline from 56.8 to 52.0 mL/min/1.73 m². Similar patterns were observed in the IPTW-weighted (**Figure 4H**) and OW-weighted cohorts (**Figure 4I**). Across all three analytical approaches, eGFR remained relatively stable in both groups, and no significant differences in temporal trends were observed between the finerenone and control groups (all P>0.05), suggesting that finerenone use did not adversely affect renal function.

### Safety outcomes

Safety analysis revealed that the incidence of hyperkalemia was lower in the finerenone group than in the control group. When analyzed by patient number, the incidence of serum potassium >5.5 mmol/L was 3.77% (2/53) in the finerenone group versus 11.1% (9/81) in the control group, without a statistically significant between-group difference (P = 0.199) (**Table 4**). The incidence of serum potassium >5.0 mmol/L was 20.8% (11/53) in the finerenone group versus 37.0% (30/81) in the control group, without a statistically significant between-group difference (P = 0.071). When analyzed by episode count, the incidence of serum potassium >5.5 mmol/L episodes did not differ significantly between groups (P = 0.615), and the incidence of serum potassium >5.0 mmol/L episodes also did not differ significantly between groups (P = 0.059). All hyperkalemia episodes were mild in severity, and serum potassium levels returned to normal ranges after symptomatic intervention, with no severe adverse events reported.

**Table 4:** Comparison of hyperkalemia occurrence between the two groups.

	Total (N = 134)	Control group (N = 81)	Finerenone group (N = 53)	p.overall
Serum potassium >5.5 mmol/L	11 (8.21%)	9 (11.1%)	2 (3.77%)	0.199
Serum potassium >5.0 mmol/L	41 (30.6%)	30 (37.0%)	11 (20.8%)	0.071
Number of episodes of serum potassium >5.5 mmol/L				0.615
0	123 (91.8%)	72 (88.9%)	51 (96.2%)	
1	8 (5.97%)	6 (7.41%)	2 (3.77%)	
3	1 (0.75%)	1 (1.23%)	0 (0.00%)	
4	2 (1.49%)	2 (2.47%)	0 (0.00%)	
Number of episodes of serum potassium >5.0 mmol/L				0.059
0	93 (69.4%)	51 (63.0%)	42 (79.2%)	
1	12 (8.96%)	10 (12.3%)	2 (3.77%)	
2	11 (8.21%)	7 (8.64%)	4 (7.55%)	
3	7 (5.22%)	7 (8.64%)	0 (0.00%)	
4	11 (8.21%)	6 (7.41%)	5 (9.43%)	

## Discussion

This study suggests that finerenone is associated with sustained graded improvement in 24h-UTP in patients with T2DM and CKD with baseline proteinuria >1 g/d, a population at particularly high risk for rapid disease progression and cardiovascular complications (2, 3). The magnitude of proteinuria reduction observed-approximately 40% from baseline at 12 months-aligns with established thresholds for clinically meaningful renoprotection (4). Notably, the graded improvement endpoint used in this study captures clinically relevant transitions in risk stratification, where each downgrade in proteinuria stage reflects a substantial reduction in the hazard of CKD progression and ESRD (23).

In our cohort, persistent proteinuria was observed despite varying levels of background RASi and SGLT2i use, representing an unmet clinical need. Our findings raise the hypothesis that finerenone may address this therapeutic gap: even in patients with moderate-to-heavy baseline proteinuria., the addition of finerenone conferred a significantly higher probability of sustained graded proteinuria improvement (52.8% vs. 33.3%) and shortened the time to improvement. This is particularly noteworthy given that the FIDELIO-DKD and FIGARO-DKD trials primarily enrolled patients with microalbuminuria to macroalbuminuria (16, 17), leaving limited evidence for those with more substantial proteinuria.

The proteinuria-lowering efficacy of finerenone observed in this study is mechanistically consistent with the central role of MR overactivation in diabetic kidney injury progression (32). Our finding that patients with higher baseline proteinuria (>3.5 g/d) showed a numerically higher point estimate for finerenone-associated proteinuria improvement (OR 3.32, 95% CI 1.15–9.57) is consistent with the hypothesis that upregulated MR expression in the setting of significant proteinuria creates a self-perpetuating cycle amenable to MR blockade (33). However, because the interaction test for baseline proteinuria subgroups was not statistically significant (P = 0.667), this observation should be interpreted as hypothesis-generating rather than as evidence of differential treatment efficacy. The sustained and cumulative reduction in 24h-UTP observed over 12 months-without compensatory eGFR decline- is consistent with the hypothesis that finerenone may interrupt this inflammatory-fibrotic cascade in a manner distinct from merely inducing hemodynamic changes. Prospective mechanistic studies are needed to validate this interpretation. Recent real-world evidence further supports the additive benefit of finerenone when combined with SGLT2i therapy (34, 35). In a large Israeli cohort of patients with T2DM and CKD already receiving SGLT2i and/or GLP-1 RA therapy, add-on finerenone achieved an additional ~51% reduction in albuminuria, with the magnitude of reduction correlating with baseline albuminuria severity (8). Consistent with these findings, recent retrospective observational studies from China (36) and Japan (37) have also demonstrated the efficacy and safety of finerenone in real-world DKD populations, further supporting its clinical utility beyond landmark trial settings.

In this study, serum potassium levels remained within the normal range in both groups throughout follow-up, with no significant between-group differences in potassium trajectories (all P>0.05). Although the numerical incidence of hyperkalemia was lower in the finerenone group, this observation must be interpreted with considerable caution. The finerenone group had significantly higher baseline eGFR (72.46 vs. 37.31 mL/min/1.73 m², P<0.001) and higher use of both RASis (66.0% vs. 44.4%, P = 0.023) and SGLT2is (79.2% vs. 44.4%, P<0.001). Since better preserved kidney function enhances renal potassium excretion capacity, and SGLT2 inhibitors have been independently shown to reduce hyperkalemia risk through enhanced distal tubular potassium delivery and secretion, the lower observed hyperkalemia incidence in the finerenone group may be substantially confounded by these baseline advantages rather than reflecting an inherent safety advantage of finerenone. Therefore, the present data cannot support a definitive conclusion regarding the comparative safety of finerenone in this setting. The stability of eGFR over 12 months in both groups further suggests that finerenone’s proteinuria reduction was not achieved at the expense of acute hemodynamic compromise.

This study included 134 patients with T2DM and CKD, all exhibiting moderate-to-heavy proteinuria (24h-UTP >1 g/d) and receiving standard treatment including RASis and/or SGLT2is. Baseline characteristics revealed that the finerenone group had relatively higher eGFR levels, likely reflecting a selection bias in clinical practice where physicians may be more inclined to prescribe finerenone to patients with relatively preserved renal function. However, after propensity score weighting to balance baseline characteristics, the association between finerenone use and sustained graded improvement in 24h-UTP remained statistically significant, and this association was independent of baseline renal function, proteinuria severity, and concomitant medications in the fully adjusted model. This finding strongly suggests that the proteinuria-lowering efficacy of finerenone is robust in this high-risk population of patients with T2DM and CKD who already exhibit moderate-to-heavy proteinuria.

From a clinical perspective, this study employed sustained graded improvement in proteinuria as the primary endpoint rather than simple absolute quantitative changes. This design was based on the following consideration: patients with moderate-to-heavy proteinuria (>1 g/d) are already at high risk for CKD progression and cardiovascular events, and any downgrade in proteinuria level (e.g., from >3.5 g/d to 1-3.5 g/d, or from 1-3.5 g/d to <1 g/d) represents a substantial reduction in risk stratification (4). In this study, proteinuria in the finerenone group decreased by 40% from baseline at 12 months, and Kaplan-Meier analysis showed a significantly shorter time to sustained graded improvement (Log-rank P = 0.026–0.033). In this population already at high risk, achieving a “downgrade” in proteinuria carries important clinical translational value-it reflects not only alleviation of glomerular injury but also predicts delayed CKD progression and improved long-term cardiorenal prognosis (2–4).

Furthermore, longitudinal analysis revealed that the finerenone group consistently demonstrated lower 24h-UTP ratios relative to baseline at all follow-up timepoints compared with the control group, with the between-group difference increasing over time, suggesting that the proteinuria-lowering effect of finerenone is sustained and cumulative. Meanwhile, eGFR and serum potassium levels remained stable in both groups during follow-up, with no significant differences in temporal trends between groups, indicating that finerenone effectively reduces proteinuria without adversely affecting renal function or electrolyte balance, further supporting its favorable safety profile. Subgroup analyses suggested that male patients, those receiving concomitant SGLT2i therapy, and individuals with higher baseline proteinuria (>3.5 g/d) may derive greater benefit from finerenone treatment, although the interaction tests did not reach statistical significance and these observations require cautious interpretation and further validation in larger samples.

Regarding safety, the incidence of hyperkalemia, defined as serum potassium >5.5 mmol/L and >5.0 mmol/L, was numerically lower in the finerenone group than in the control group. However, as detailed above, this difference likely reflects the confounding effects of higher baseline eGFR and greater SGLT2i/RASi use in the finerenone group rather than a drug-specific safety advantage. It is important to note that both the FIDELIO-DKD and FIGARO-DKD trials reported hyperkalemia as a significant adverse effect of finerenone, with markedly higher rates in the finerenone groups than in the placebo groups (16, 17). The lower hyperkalemia incidence observed in our study should therefore not be interpreted as evidence of superior safety. Nevertheless, the fact that no severe hyperkalemia events requiring emergency intervention occurred in either group is noteworthy, as it suggests that with individualized dose selection and regular monitoring, finerenone can be administered without life-threatening hyperkalemia in carefully selected patients.

Several limitations should be acknowledged. First, this was a retrospective observational study without randomization, which is inherently susceptible to treatment selection bias and confounding. Although propensity score weighting (IPTW and OW) was applied to balance all measured baseline covariates between groups, this method can only adjust for observed confounders and cannot eliminate bias from unmeasured or unknown confounders such as blood pressure control levels, dietary sodium intake, medication adherence, and physician prescribing preferences. Moreover, although the propensity score covariate set was substantially expanded to 16 variables (including age, sex, smoking history, alcohol consumption, BMI, history of hypertension, baseline fasting blood glucose, LDL-C, HDL-C, triglycerides, total cholesterol, HbA1c, baseline 24h-UTP, baseline eGFR, baseline SGLT2i use, and baseline RASi use), residual imbalance (SMD >0.1) persisted for several key variables (baseline eGFR and SGLT2i use) after IPTW weighting. While the OW method achieved better balance and the regression models incorporated additional adjustment for residual confounders, we cannot exclude the possibility that residual selection bias or unmeasured confounding contributed to the observed associations. The direction and magnitude of any such bias remain uncertain. Therefore, the observed associations should not be interpreted as causal, and the findings remain hypothesis-generating. Second, the single-center design limits the generalizability of our findings to broader populations with different demographic and clinical characteristics. Third, the follow-up duration of 12 months was relatively short, and long-term efficacy and safety, including hard renal endpoints such as sustained eGFR decline or progression to end-stage renal disease, require further validation. Fourth, the study population comprised patients with T2DM and CKD with proteinuria >1 g/d, and the findings may not be directly generalizable to patients with lesser degrees of proteinuria or those with non-diabetic CKD. Fifth, the observed treatment effect sizes, particularly in the propensity score-weighted analyses, were relatively modest. The *post-hoc* power analysis indicated that the study had approximately 60% power to detect the observed effect size (OR = 2.24) with the original sample size, and approximately 47% power with the IPTW-weighted effective sample size. The minimum detectable OR with 80% power was 2.73, exceeding the observed OR of 2.24. This suggests that the study may be underpowered for detecting modest treatment effects, and the observed effect estimate may be subject to overestimation. The relatively wide 95% confidence intervals further reflect limited precision of the effect estimates. Future prospective, multicenter, large-scale, randomized controlled trials with extended follow-up and larger sample sizes are warranted to obtain more precise estimates of the treatment effect and to confirm these findings. Sixth, the proportion of patients receiving guideline-recommended RASi and SGLT2i therapy was suboptimal (approximately 53% and 58%, respectively), reflecting real-world treatment barriers. Seventh, the safety comparison between groups is subject to substantial confounding. The finerenone group had significantly better baseline kidney function and higher use of medications known to reduce hyperkalemia risk (particularly SGLT2is), which may have masked the true hyperkalemia risk associated with finerenone. The observed lower hyperkalemia incidence in the finerenone group should therefore not be interpreted as evidence of a favorable safety profile; rather, it may reflect a more favorable baseline risk profile for potassium-related adverse events.

The findings of this study are observational and hypothesis-generating. While the graded proteinuria improvement and stable eGFR over 12 months are encouraging, they do not establish finerenone’s efficacy in altering long-term disease progression. The observed association between finerenone use and proteinuria reduction warrants further investigation in prospective trials to determine whether finerenone confers clinically meaningful cardiorenal protection in this population. Future prospective, multicenter randomized controlled trials with extended follow-up are warranted to confirm these findings and further elucidate the optimal timing and patient selection for finerenone initiation.

## Conclusions

In this retrospective cohort of patients with T2DM and CKD with moderate-to-heavy proteinuria (24h-UTP >1 g/d), finerenone use was associated with a higher probability of sustained graded improvement in 24h-UTP and a shorter time to improvement, with no significant increase in hyperkalemia risk over 12 months. These findings are hypothesis-generating and suggest that finerenone may be a candidate for further prospective evaluation as an adjunctive therapy for persistent proteinuria in this high-risk population. Whether the observed proteinuria reduction translates into improved long-term cardiorenal outcomes requires confirmation in randomized controlled trials.

## Data Availability

The original contributions presented in the study are included in the article/supplementary material. Further inquiries can be directed to the corresponding author/s.
